# NANOG helps cancer cells escape NK cell attack by downregulating ICAM1 during tumorigenesis

**DOI:** 10.1186/s13046-019-1429-z

**Published:** 2019-10-16

**Authors:** Kotaro Saga, Jinhee Park, Keisuke Nimura, Norihiko Kawamura, Airi Ishibashi, Norio Nonomura, Yasufumi Kaneda

**Affiliations:** 1 Division of Gene Therapy Science, Osaka University Graduate School of Medicine, 2-2 Yamada-oka, Suita, Osaka, 565-0871 Japan; 2 Department of Urology, Osaka University Graduate School of Medicine, Suita, Osaka, 565-0871 Japan

**Keywords:** NANOG, ICAM1, NK cell, Tumorigenesis

## Abstract

**Background:**

At the beginning of tumorigenesis, newly born cancer cells must successfully avoid attack by the immune system. Although most abnormal cells are efficiently identified and destroyed by the immune system, particularly by NK cells, the molecular mechanisms by which newly born cancer cells evade NK cell surveillance are not fully understood.

**Methods:**

NK cell resistance of highly tumorigenic population of human prostate cancer (PCa) cells were confirmed by xenograft in SCID mice with or without NK cell neutralization. The mechanisms by which the tumorigenic PCa cells evaded NK cell attack were investigated by RNAseq, ChIPseq, generation of several transformants and xenograft in SCID mice.

**Results:**

Here, we show that PCa cells have a strengthened ability to escape NK cell attack due to NANOG, a pluripotent-related transcription factor, mediating the repression of ICAM1, a cell adhesion molecule, during tumorigenesis. Mechanistically, NANOG directly binds to the region upstream of *ICAM1*. As the binding between NANOG and the upstream *ICAM1* region increases, p300 binding to this region is diminished, resulting in decreased ICAM1 expression. High NANOG expression confers PCa cells the ability to resist NK cell attack via the repression of ICAM1. Consistent with these results, low *ICAM1* expression is significantly correlated with a high recurrence rate in patients with PCa.

**Conclusions:**

Our findings indicate that repression of ICAM1 is a critical mechanism by which cancer cells evade attack from NK cells during tumorigenesis. These results suggest a pivotal role of NANOG in establishing a gene expression profile for escaping the immune system.

## Background

Tumorigenesis is continuously monitored by the immune system, and most newly born cancer cells are eliminated by anticancer immune responses [1, 2]. However, some newly born cancer cells evade immune surveillance, defined as cancer-initiating cells (CICs), and thus exhibit tumorigenic potential, resulting in tumor formation. As the tumor mass increases, chemokines secreted from cancer cells attract various host-derived immunosuppressive cells (e.g., regulatory T cells [3], myeloid-derived suppressor cells [4], tumor-associated macrophages [5] and tumor-associated neutrophils [6]) into tumors. Thus, tumor tissues eventually consist of heterogeneous cell populations that include numerous cancer cells and various host-derived immunosuppressive cells [7]. These heterogeneous cells establish an immunosuppressive environment in the tumor tissue by maintaining high cytokine levels [8–12], promoting the production of cancer-derived exosomes [13] and exerting immunosuppressive effects on intratumoral host-derived immunosuppressive cells [14], thus protecting cancer cells from immune cell attack. On the other hand, during the early phase of tumorigenesis, CICs and other cancer cells derived from CICs establish a poor immunosuppressive environment due to insufficient cytokine secretion, exosome production and host-derived immunosuppressive cell attraction. Therefore, these cancer cells require a distinct anticancer immune escape system to allow tumor tissue formation from the tumor tissue-mediated immunosuppressed environment. However, the molecular mechanisms by which CICs evade anticancer immune surveillance during the initial stage of tumor formation via the establishment of an immunosuppressive environment remain incompletely understood.

CIC-like phenotypic cancer cells, which exhibit high tumorigenic activity, have been identified in various tumor tissues and cultured cancer cells [15–19] and have a distinctive gene expression profile unlike that of normal cancer cells [20, 21]. In particular, the upregulation of stem cell factors, e.g., NANOG, OCT4 and SOX2, are distinguishing characteristics of CIC-like cells, and these transcription factors are important for maintenance of the CIC-like phenotype [22]. However, the mechanisms by which these transcription factors provide cancer cells the ability to evade anticancer immune responses remain unknown.

Herein, we show that the NANOG-mediated repression of ICAM1 is a critical mechanism underlying the ability of cancer cells to escape natural killer (NK) cell attack during the initial stage of prostate cancer (PCa) formation.

## Methods

### Cell culture

Human PCa cells (DU145, PC3, 22Rv1) were purchased from the American Type Culture Collection (Rockville, USA) and maintained in Dulbecco’s Modified Eagle’s Medium (DMEM) (Nacalai Tesque Inc., Tokyo, Japan). MTA cells were purchased from Japanese Collection of Research Bioresources Cell Bank (Ibaraki, Japan) and maintained in RPMI-1640 medium (Nacalai Tesque). Both DMEM and RPMI-1640 medium were supplemented with 10% fetal bovine serum (FBS) (Biowest, Nuaillé, France), 100 U/mL penicillin and 0.1 mg/mL streptomycin (Penicillin–Streptomycin Mixed Solution) (Nacalai Tesque). These cells were incubated at 37 °C and 5% CO_2_.

### Sphere-forming culture

Spheres of DU145 cells were formed as previously described [23]. Briefly, DU145 cells were plated on ultralow attachment culture dishes (Corning, NY, USA) (1 × 10^3^ cells/well in 6-well plates and 1 × 10^5^ cells/dish in 10 cm dishes) and cultured in DMEM/F-12 (Gibco, NY, USA) supplemented with B27 (Gibco), 4 μg/mL insulin (Sigma, MO, USA), 20 ng/mL epithelial growth factor (EGF; Gibco), and 20 ng/mL basic fibroblast growth factor (bFGF) (ORF, Kopavogur, Iceland) for 10 days at 37 °C and 5% CO_2_.

### Plasmid construction

Human NANOG cDNA was amplified from PC3 cDNA, and GFP-NANOG was generated by connecting NANOG cDNA to the 3′-terminus of EGFP cDNA. Human ICAM1 cDNA was purchased from R&D Systems (Minnesota, USA). GFP and GFP-NANOG cDNA were introduced into the CAGIpuro vector, and ICAM1 cDNA was introduced into the CAGIneo Gateway vector. pX330 (Addgene, MA, USA) was treated with BbsI, and DNA oligos comprising a gRNA sequence (GCTCTGTTCCCAGGTGAGTC and GCTATTCAAACTGCCCTGAT) were inserted into the BbsI site.

### Generation of stable transformants and ICAM1KO-DU145 cells

Plasmid DNA (1 μg) was transfected into suspended DU145, PC3 or 22Rv1 cells (1 × 10^5^ cells) using the NENO system (Invitrogen) (voltage; 1150, width; 30, pulse number; 2). Transfected DU145 cells were cultured with 1.0 μg/mL puromycin (Nacalai Tesque) or 2 mM G418 (Nacalai Tesque), while transfected PC3 and 22Rv1 cells were respectively cultured with 4.0 μg/mL and 0.5 μg/mL puromycin. Single colonies were selected and individually cultured. pX330 including the target gRNA sequence (1 μg) and pPGK-puro (0.5 μg) were transfected into suspended DU145 cells (1 × 10^5^) using the NENO system; the DU145 cells were then cultured with 1.0 μg/mL puromycin for 1 week and then cultured without puromycin. Single colonies were selected and individually cultured. Genotypes were identified by PCR, which was performed using Q5 high-fidelity DNA polymerase (New England Biolabs Inc. (NEB), MA, USA).

### Cell proliferation assay

WT-, GFP-, and GN-DU145 cells as well as WT-, GFP-, and GN-PC3 cells were seeded on 6-well plates at 1 × 10^5^ cells/well. These cells were cultured, and the cell numbers were counted every other day.

### Western blot analysis

Cell lysates were subjected to SDS-PAGE, and separated proteins were transferred onto Immobiron-P Transfer Membranes (Merck Millipore, Tokyo, Japan). To detect proteins, the anti-NANOG (Cell Signaling Technology (CST), MA, USA; #4903), anti-ICAM1 (CST; #4915), anti-GFP (CST; #2956) and anti-βactin (Sigma; A5441) primary antibodies were used. Enhanced chemiluminescence (ECL) horseradish peroxidase-conjugated donkey anti-rabbit IgG (GE Healthcare Japan, Tokyo, Japan) was used as a secondary antibody for the detection of NANOG, ICAM1 and GFP, and ECL horseradish peroxidase-conjugated sheep anti-mouse IgG (GE Healthcare) was used as a secondary antibody for the detection of βactin. Signals were detected using Chemi-Lumi One (Nacalai Tesque) or Chemi-Lumi One Super (Nacalai Tesque) and an ImageQuant LAS 4000 mini system (GE Healthcare).

### Real-time RT-PCR

Total RNA was isolated from each cell using the ISOGEN kit (Nippon Gene, Tokyo, Japan), and cDNA was generated from total RNA using a High Capacity cDNA Reverse Transcription Kit (Applied Biosystems Inc., CA, USA). NANOG and ICAM1 expression was quantified via real-time reverse transcriptase PCR (RT-PCR) using THUNDERBIRD SYBR qPCR Mix (TOYOBO, Tokyo, Japan). As a control, 18S ribosomal RNA was quantified in the same manner.

### Assaying NK cell resistance in vitro

DU145 and PC3 cells were seeded on 6-well plates (1 × 10^5^ cells/well) and incubated for 4 h to allow adhesion to the plate bottom. MTA cells were added to each well at a 1:1 ratio (1 × 10^5^ cells), and the cells were cocultured for 48 h. Anti-ICAM1 Ab (CST; #4915) was added to culture medium at 1/1000 dilution for ICAM1 neutralization. After coculture, floating cells, including MTA and dead cancer cells, were eliminated by washing with PBS, and adherent surviving cancer cells were measured by the MTS assay using the CellTiter 96 Aqueous One Solution Cell Proliferation Assay kit (Promega, WI, USA).

### Assaying tumor formation in vivo

DU145 cells and their derivatives (1 or 5 × 10^5^ cells) were intradermally transplanted onto the backs of SCID mice (CLEA Japan, Suita, Japan). For NK cell neutralization, an anti-asialo GM1 antibody (Wako, Osaka, Japan; 200 μg/200 μL PBS) was intraperitoneally administered to SCID mice before and after (− 1, 0, 1, 2, 4, 6, 9, 12, 15 and 19 days) cancer cell transplantation. As a control, normal rabbit IgG (Sigma) was administered in the same manner. Tumor growth was monitored by periodically measuring tumor volume using calipers; tumor volume was calculated according the following formula: tumor volume (mm)^3^ = maximal length (mm) x (perpendicular width) (mm)^2^ / 2.

### RNAseq

RNAseq libraries were prepared using the NEBNext Poly(A) mRNA Magnetic Isolation Module (NEB), the NEBNext Ultra RNA Library Prep Kit for Illumina (NEB) and the NEBNext Multiplex Oligos for Illumina (NEB) and sequenced by Illumina.

### ChIPseq

Crosslink ChIP: WT-, GFP- and GN-DU145 cells (2 × 10^7^) were fixed with 1% formaldehyde (Sigma) and sonicated using the Sonifier SFX550 instrument (BRANSON, CT, USA) to induce chromatin fragmentation. Anti-NANOG (CST; #5232) and anti-p300 (Active Motif, CA, USA; #61401) Abs were bound to Protein G Dynabeads (Thermo Fisher Scientific, MA, USA), and these conjugates were used for ChIP. Native ChIP: Nuclear fractions of WT-, GFP- and GN-DU145 cells (1 × 10^6^) were treated with 1200 U MNase (Takara, Shiga, Japan) for chromatin fragmentation. The anti-H3K27Ac Ab (Abcam, Cambridge, UK; ab4729) was bound to Protein G-agarose (Merck Millipore), and the conjugate was used for ChIP. The ChIPseq library was prepared using the NEBNext Ultra II DNA Library Prep Kit for Illumina (NEB) and the NEBNext Multiplex Oligos for Illumina. ChIPseq libraries were sequenced by Illumina.

### ChIP qPCR

ChIP was performed according to the manufacturer’s instructions (SimpleChIP Enzymatic Chromatin IP Kit (#9003); CST). ChIP samples of WT-, GFP, and GN-DU145 cells were amplified by primers targeting the ICAM1 promoter region (ACCGAGCCTGGCCCCAA and CTGACCTCGTGTTCCACCTGC) and quantified using THUNDERBIRD SYBR qPCR Mix.

### Clinical dataset analysis

The ICAM1 expression levels in localized PCa and CRPC tissues were analyzed using previously published microarray-based gene expression data [24]. cBioPortal [25] was used to analyze the correlation between ICAM1 expression levels and progression-free rates in a prostate adenocarcinoma dataset (TCGA, Provisional). To assess the progression-free rate after total prostatectomy, data harvested from patients in the chemotherapy group before surgery, patients in the radiotherapy group after surgery and patients with an unknown follow-up period and prognosis were eliminated from the original dataset. The final dataset was separated into two groups depending on the ICAM1 expression level, and levels in the top and bottom 50% were respectively categorized into the ICAM1-high and ICAM1-low groups, respectively.

## Results

### Sphere-forming cancer cells have tumorigenic potential and escape from NK cell attack

In immunodeficient mice, PCa spheres exhibit many CIC-like characteristics and a higher tumorigenic potential than adherent-cultured cancer cells [15, 16, 26, 27]. To confirm the phenotype of sphere-forming cancer cells, sphere-forming human PCa cell lines (DU145) were generated by culturing cells in serum-free medium on a low adherent culture dish. Although DU145 cells appeared in a cobblestone form when cultured in normal adherent medium (Additional file 1: Figure S1A, left), they formed floating spheres within 10 days under this culture condition (Additional file 1: Figure S1A, right). To compare the tumorigenicities of both forms of DU145 cells (sphere-forming DU145 cells (SP-DU145) and adherent-cultured DU145 cells (AD-DU145)), 1 × 10^5^ cells of each form were intradermally inoculated onto the backs of SCID mice (Additional file 1: Figure S1B). SP-DU145 cells exhibited tumorigenic potential, while AD-DU145 cells did not, consistent with previous reports (Additional file 1: Figure S1C). Tumor formation assays of sphere-forming cancer cells were carried out using SCID or NOD/SCID mice in this experiment and in previous reports [15, 16, 26, 27]. These immunodeficient mice lack T and B cells but maintain NK cell activity [28, 29]. Therefore, transplanted sphere-forming cancer cells must escape from NK cell attack by themselves during the initial stage before a tumor tissue-mediated immunosuppressive environment is established.

**Fig. 1:**
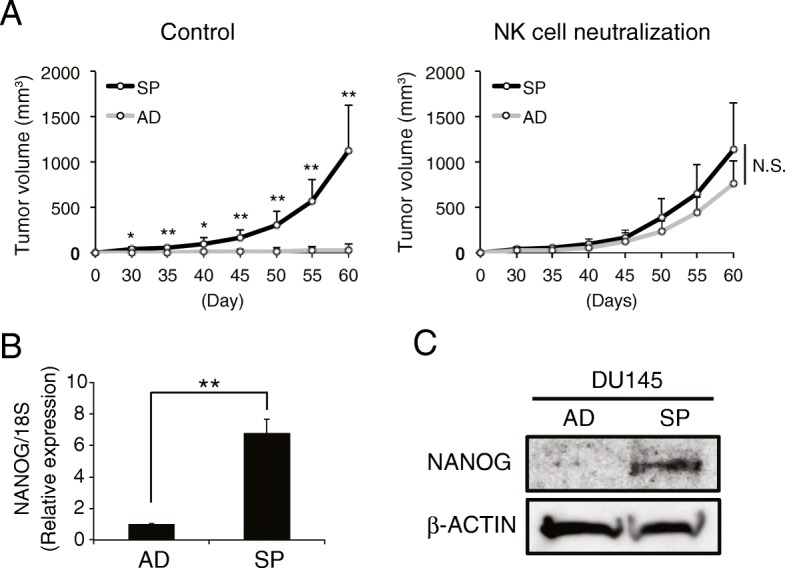
Sphere-forming DU145 cells exhibit tumorigenic potential by escaping from NK cell attack. (**a**) AD- and SP-DU145 cells were transplanted onto SCID mice treated with an anti-asialo GM1 Ab for NK cell neutralization or control IgG. The respective tumor volumes were measured over time (*n* = 4, *P < 0.05, **P < 0.01, Student’s t test). (**b** and **c**) The NANOG expression levels in AD- and SPDU145 cells were compared by real-time RT-PCR (**b**) and western blot (**c**) analyses. Real-time RT-PCR was performed in triplicate. (**P < 0.001, Student’s t test)

Next, we investigated the relationship between the tumorigenic potential and NK cell sensitivity of DU145 cells. SCID mice were continually injected with an anti-asialo GM1 antibody (NKneut-SCID mice) via the intraperitoneal route to inhibit NK cell activity; as a control, normal rabbit IgG was injected into SCID mice in the same manner (Cont-SCID mice) (Additional file 1: Figure S1D). These injections were performed for 19 days after the transplantation of cancerous cells to neutralize NK cells from the initial stage through the formation of tumor tissue. AD- and SP-DU145 cells were intradermally transplanted onto the backs of these SCID mice, and their tumorigenic potentials were then observed separately (Additional file 1: Figure S1D). In Cont-SCID mice, SP-DU145 cells formed tumors in all mice, while AD-DU145 cells exhibited very low tumorigenicity. However, both SP-DU145 cells and AD-DU145 cells exhibited tumorigenicity in NKneut-SCID mice **(**Fig. 1 A**)**. These results suggested that the tumorigenic potential of DU145 cells was promoted by the acquisition of NK cell resistance via sphere formation.

### NANOG overexpression promotes escapability from NK cell attack

Several transcription factors contribute to the maintenance of CIC-like characteristics, which include high tumorigenic potential and some malignant phenotypes [30]. In particular, NANOG is highly expressed in CIC-like PCa cell lines [31, 32], and our group has revealed that NANOG is important for the tumorigenesis of PCa cells [33]. In fact, SP-DU145 cells exhibited higher NANOG expression than AD-DU145 cells **(**Fig. 1 B and C**)**. Moreover, tumors comprised of highly malignant PCa tissue had more CIC-like cells than those comprised of low malignant PCa tissue [34], and NANOG expression was higher in highly malignant PCa tumors (Grison score (GS) 9) than in low malignant PCa tumors (GS6) (Additional file 1: Figure S2). Therefore, these results suggest a link between NANOG expression and NK cell resistance in PCa.

**Fig. 2:**
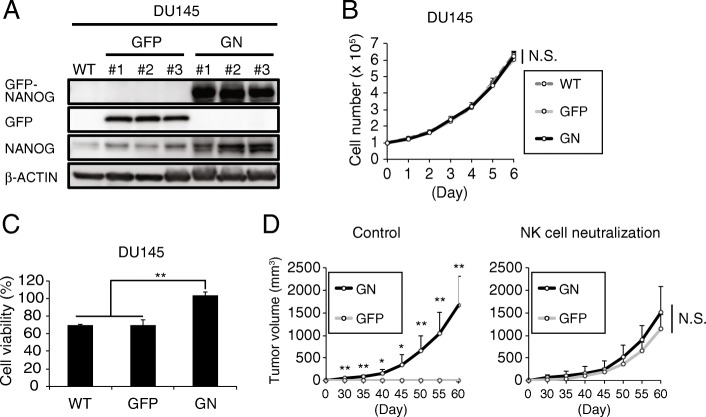
NANOG overexpression induces tumorigenic potential by allowing escape from NK cell attack. (**a**) Western blot analyses of WT-, GFP- and GN-DU145 cells. (**b**) The proliferation rates of DU145 cells (WT, GFP, and GN) were compared (*n* = 3, Tukey-Kramer test). (**c**) The cell viabilities of DU145 cells (WT, GFP, and GN) were measured after performing the NK cell resistance assay in vitro (n = 3, *P < 0.005, **P < 0.001, Tukey-Kramer test). (**d**) GFP- and GN-DU145 cells were transplanted onto SCID mice treated with an anti-asialo GM1 Ab or control IgG. Respective tumor volumes were measured for up to 60 days after the transplantation (n = 4, *P < 0.05, **P < 0.01, Student’s t test)

To investigate the relationship between NANOG expression and NK cell resistance in PCa, GFP-NANOG-overexpressing DU145 (GN-DU145) and PC3 (GN-PC3) cells were generated (Fig. 2 A **and** Additional file 1: Figure S3A); GFP-overexpressing DU145 (GFP-DU145) and PC3 (GFP-PC3) cells were generated as controls. GN-DU145 and GN-PC3 cells exogenously expressed GFP-NANOG and exhibited increased intrinsic NANOG expression levels compared with those in their respective wild-type (WT) and GFP groups (Fig. 2 A **and** Additional file 1: Figure S3A). Moreover, the proliferation rates of GN-DU145 and GN-PC3 cells equaled those of their respective controls (Fig. 2 B **and** Additional file 1: Figure S3B). Next, we assessed whether NANOG overexpression influenced NK cell resistance. NK cells have the ability to lyse myeloid leukemia cells (K562) when the two cell types are cocultured [35]. Because NK-like T cell leukemia/lymphoma (MTA) cells exhibited NK cell-like lysis activity against cocultured K562 cells despite exhibiting little activity toward another leukocyte cell line (M1 macrophage cell line) (Additional file 1: Figure S3C), MTA cells were used to assay in vitro resistance against NK cell attack. WT-, GFP- and GN-DU145 cells and WT-, GFP- and GN-PC3 cells were cultured with MTA cells for 48 h, and the cell viabilities of DU145 and PC3 cells were measured by the MTS assay (Fig. 2C **and** Additional file 1: Figure S3D). The survival rates of WT-DU145, GFP-DU145, WT-PC3, and GFP-PC3 cells were decreased to 60 ~ 80%, while GN-DU145 and GN-PC3 cells were hardly attacked by MTA cells. Moreover, MTA-treated apoptotic (AnnexinV+) DU145 cells were measured by FACS, and GN-DU145 had fewer AnnexinV+ population than WT-DU145 (Additional file 1: Figure S3E). Furthermore, GN-DU145 also exhibited higher resistance against human NK cell from healthy donor than control group (Additional file 1: Figure S3F). Next, GFP- and GN-DU145 cells were intradermally transplanted onto the backs of the NKneut- and Cont-SCID mice, and their respective tumorigenic potentials were observed **(**Fig. 2 D**)**, revealing that GFP-DU145-derived tumors were formed in only NKneut-SCID mice. However, GN-DU145 cells were able to generate tumors in both types of SCID mice. Therefore, NANOG expression in PCa cells promoted escape from NK cell attack.

### NANOG overexpression induced NK cell resistance by downregulating ICAM1

**Fig. 3:**
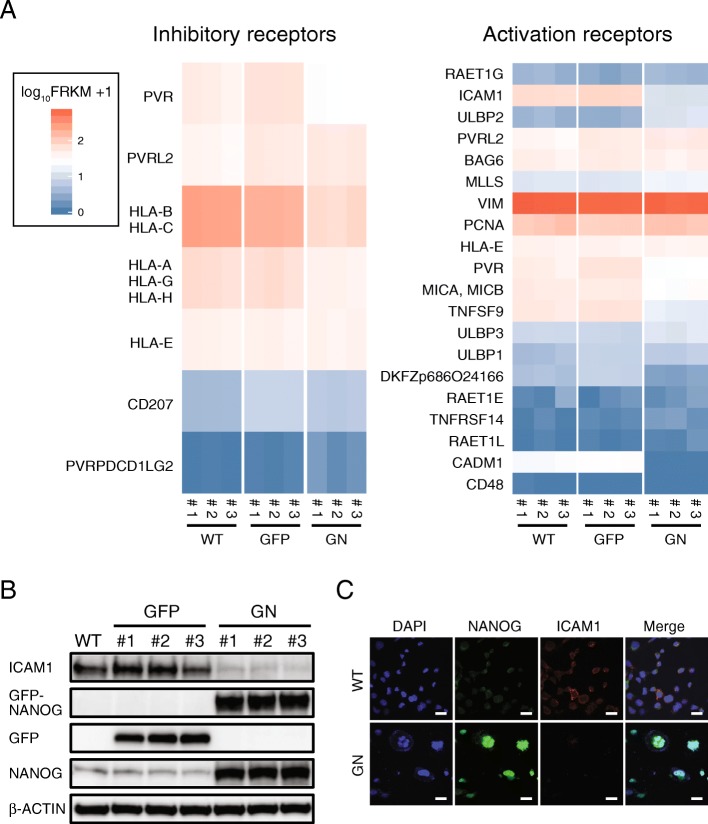
NANOG overexpression downregulates ICAM1 expression. (**a**) RNAseq analysis of WT-, GFP-, and GN-DU145 cells. The expression levels of NK cell inhibitory receptors and activation receptors were compared among these cells. (**b**) ICAM1 expression was detected in WT-, GFPand GN-DU145 cells by western blot. (**c**) ICAM1 localization was detected in WT- and GN-DU145 cells by immunofluorescence staining

Tumorigenic cancer cells must escape from immune cells individually because a few cells will unlikely be insufficient for successfully establishing an immune suppressive environment from the initial stage all the way through tumor tissue formation. Tumorigenic cancer cells might evade NK cell attack by regulating membrane surface proteins [36], and we hypothesized that NANOG upregulates NK cell inhibitory factors or downregulates NK cell activation factors in PCa cells. The gene expression profiles of these factors were analyzed in WT-, GFP- and GN-DU145 cells by RNAseq **(**Fig. 3 A**,** Additional file 1**:** Figures. S4A and S5A). Upregulated inhibitory factors and immune check point ligands were not detected in GN-DU145 cells **(**Fig. 3 A left and Additional file 1: Figure S5A**)**, while several activation factors were significantly downregulated in these cells **(**Fig. 3 A right). We focused on ICAM1, the gene most highly expressed in WT- and GFP-DU145 cells among genes whose expression was suppressed more than 2-fold in GN-DU145 cells compared with that in WT- and GFP-DU145 cells (both *P*-values = 0.00005). ICAM1 downregulation at the protein level was also detected in GN-DU145 cells compared with that in WT- and GFP-DU145 cells **(**Fig. 3 B**)**. Moreover, ICAM1 expression was inhibited by NANOG overexpression in another PCa cell line (22Rv1) **(**Additional file 1**:** Figure S5B). ICAM1, a type-1 transmembrane protein that is localized on the cell surface, activates NK cells via binding to LFA1 [37, 38]. ICAM1 localization was detected by immunofluorescence staining, revealing that WT-DU145 cells expressed ICAM1 on the plasma membrane, while GN-DU145 cells did not express ICAM1 **(**Fig. 3 C**)**. Taken together, these results demonstrated that NANOG overexpression downregulated ICAM1 expression in PCa cells.

**Fig. 4:**
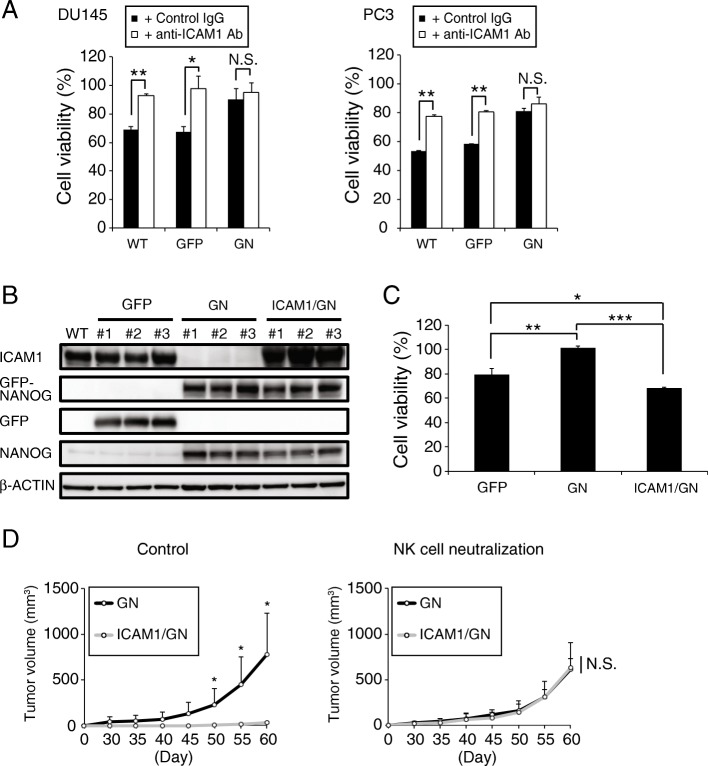
ICAM1 downregulation induces NK cell resistance (**a**) The viabilities of DU145 and PC3 cells (WT, GFP, and GN) were measured after performing the NK cell resistance assay with or without the ICAM1 neutralizing antibody in vitro (n = 3, *P < 0.005, **P < 0.0001, Student’s t test). (**b**) Generation of ICAM1-overexpressing GN-DU145 cells. (**c**) The viabilities of GFP-, GN- and ICAM1/GN-DU145 cells were measured after performing the NK cell resistance assay in vitro (n = 3, *P < 0.01, **P < 0.001, ***P < 0.0001, Tukey-Kramer test). (**d**) ICAM1/GN- and GN-DU145 cells were transplanted onto SCID mice treated with an anti-asialo GM1 Ab or control IgG. Respective tumor volumes were measured for up to 60 days after the transplantation (n = 4, *P < 0.05, Student’s t test)

Next, we investigated whether ICAM1 downregulation is responsible for the NK cell resistibility of PCa cells. The viabilities of WT-, GFP- and GN-DU145 cells and WT-, GFP- and GN-PC3 cells were measured after coculture with MTA cells treated with either an ICAM1 neutralizing antibody or a control IgG. ICAM1 neutralization significantly inhibited the cell deaths of WT and GFP-DU145 cells and WT and GFP-PC3 cells; however, the survival rates of GN-DU145 and GN-PC3 cells were not different between the groups treated with the ICAM1 antibody and the control IgG **(**Fig. 4 A**)**. Moreover, ICAM1-overexpressing GN-DU145 (ICAM1/GN-DU145) cells were generated **(**Fig. 4 B), and their resistance against MTA cells was measured by the MTS assay **(**Fig. 4 C**)**. Although GN-DU145 cells were able to escape from MTA attack, the survival rate of ICAM1/GN-DU145 cells was decreased by 60–70% after MTA attack. Furthermore, ICAM1/GN- and GN-DU145 cells were intradermally transplanted onto the backs of NKneut- and Cont-SCID mice, and their respective tumorigenic potentials were observed. GN-DU145 cells were capable of forming tumors in both types of SCID mice, while ICAM1/GN-DU145-derived tumors were not detected in Cont-SCID mice **(**Fig. 4 D**)**. Therefore, these data suggest that NANOG overexpression induced NK cell resistance in PCa cells by downregulating ICAM1.

### NANOG downregulates ICAM1 expression by inhibiting p300 recruitment

**Fig. 5:**
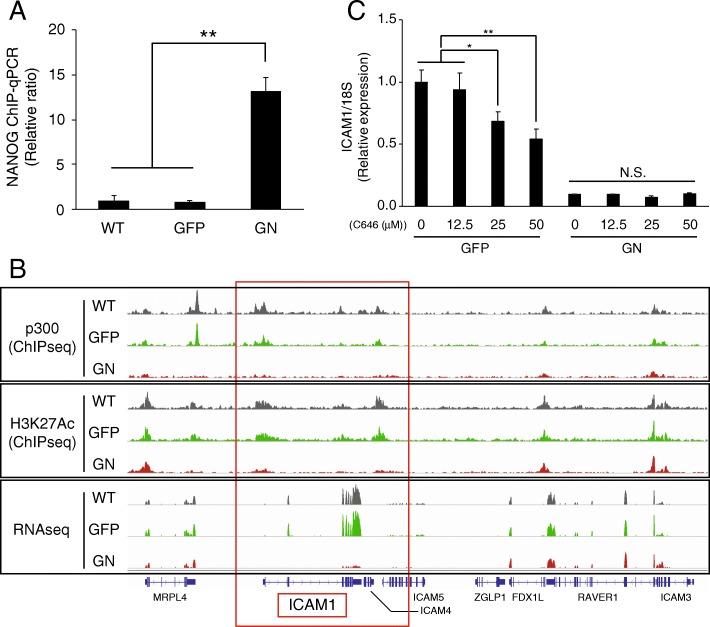
ICAM1 expression is regulated by p300 (**a**) ChIP qPCR analyses of NANOG expression in WT-, GFP-, and GN-DU145 cells (n = 3, **P < 0.0001, Tukey-Kramer test). (**b**) ChIPseq and RNAseq analyses of the region surrounding the ICAM1 gene locus in WT- (black), GFP- (green), and GN-DU145 (red) cells. (**c**) ICAM1 expression levels in GFP- and GN-DU145 cells treated with or without C646 (n = 3, *P < 0.01, **P < 0.0001, Tukey-Kramer test)

In our experiments, NANOG was shown to inhibit ICAM1 expression. It was previously reported that ICAM1 expression was downregulated by ICAM1-targeting miRNA (miR-296-3p) in PCa [39], however, NANOG overexpression did not increase miR-296-3p expression (Additional file 1: Figure S5C). To investigate how NANOG downregulates ICAM1 transcription, we assessed the ability of NANOG to bind the ICAM1 promoter region. NANOG ChIP qPCR analysis was performed to detect binding between NANOG and the ICAM1 promoter region **(**Fig. 5 A**)**, revealing that NANOG binding was significantly increased in GN-DU145 cells compared with that in WT- and GFP-DU145 cells. These results suggest that NANOG binds to the ICAM1 promoter region. Next, to investigate the mechanism by which NANOG controls ICAM1 transcription, ChIPseq analysis was performed on WT-, GFP- and GN-DU145 cells **(**Fig. 5 B and Additional file 1: Figure S4B**)**. RNAseq analysis **(**Fig. 3 A**)** showed remarkably decreased ICAM1 transcription levels in GN-DU145 cells compared with those in WT- and GFP-DU145 cells; according to these results, histone H3 lysine 27 acetylation (H3K27Ac), a known transcription activation marker, was decreased on the ICAM1 gene in GN-DU145 cells **(**Fig. 5 B**)**. H3K27 acetylation is induced by p300 histone acetyltransferase, a transcription coactivator [40]. In fact, p300 bound to the ICAM1 promoter region in WT- and GFP-DU145 cells, while significantly decreased p300 binding in this region was observed in GN-DU145 cells **(**Fig. 5 B**)**. Next, GFP- and GN-DU145 cells were cultured with a p300 inhibitor (C646); GN-DU145 cells maintained a low level of ICAM1 expression in both the presence and absence of C646, while the ICAM1 expression in GFP-DU145 cells was inhibited by C646 in a dose-dependent manner **(**Fig. 5 C**)**. Therefore, p300 contributed to the regulation of ICAM1 transcription. To exclude the possibility that an extra GFP protein fused with NANOG to inhibit the p300 association, ICAM1 expression was detected in DU145-overexpressing GFP-P2A-NANOG (GN-P2A) cells, which were divided into GFP and NANOG portions via P2A cleavage [41] (Additional file 1: Figure S5D). Because GN-P2A-DU145 cells expressed ICAM1 at low levels, like GN-DU145 cells (Additional file 1: Fig. S5D), the GFP protein likely did not inhibit the p300 association in the ICAM1 promoter region. Taken together, these results suggested that NANOG overexpression induced the NANOG association and p300 dissociation to/from the ICAM1 promoter region and consequently inhibited ICAM1 transcription.

### ICAM1 downregulation is a critical mechanism by which cells escape NK cell attack

**Fig. 6:**
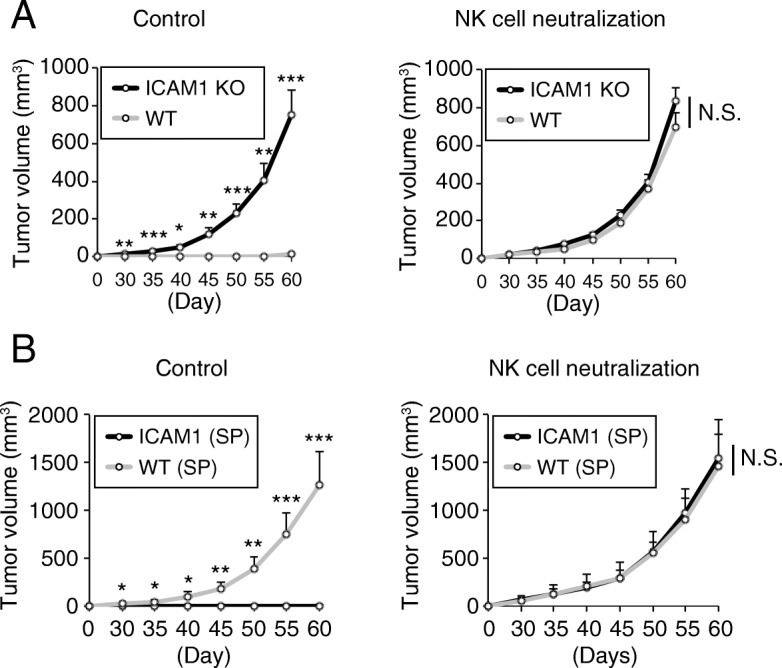
ICAM1 downregulation is an important factor underlying the high tumorigenic potential. (**a**) Adherent-cultured ICAM1KO- and WT-DU145 cells were transplanted onto SCID mice treated with an anti-asialo GM1 Ab or control IgG. Respective tumor volumes were measured for up to 60 days after the transplantation (n = 4, *P < 0.005, **P < 0.001, ***P < 0.0001, Student’s t test). (**b**) Sphere-forming ICAM1- and WT-DU145 cells were transplanted onto SCID mice treated with an anti-asialo GM1 Ab or control IgG. Respective tumor volumes were measured for up to 60 days after the transplantation (n = 4, *P < 0.05, **P < 0.01, ***P < 0.001, Student’s t test)

Up to this point, ICAM1 downregulation was shown to induce NK cell resistance in exogenous NANOG-overexpressing PCa cells. To assess the role of ICAM1 in the NK cell resistance of WT-DU145 cells not exogenously overexpressing NANOG, we attempted to manipulate ICAM1 in WT-DU145 cells. First, ICAM1 was knocked out in DU145 cells (ICAM1KO-DU145) using the CRISPR-Cas9 system **(**Additional file 1: Figure S6A), and the proliferation rate of ICAM1KO-DU145 cells was almost the same as that of WT-DU145 cells (Additional file 1: Figure S6B). Adherent-cultured WT- and ICAM1KO-DU145 cells were intradermally transplanted onto the backs of NKneut- and Cont-SCID mice, and their respective tumorigenic potentials were observed. Despite being grown in adherent culture medium, ICAM1KO-DU145 cells exhibited tumorigenic potential in both types of SCID mice, while WT-DU145 cells induced tumor formation in only NKneut-SCID mice **(**Fig. 6 A**)**. Next, ICAM1-overexpressing DU145 cells (ICAM1-DU145) were generated (Additional file 1: Figure S6C), and their proliferation rate was almost the same as that of WT-DU145 cells (Additional file 1: Figure S6D). WT- and ICAM1-DU145 cells were cultured in sphere-forming conditions, and sphere-forming WT- and ICAM1-DU145 cells were intradermally transplanted onto the backs of NKneut- and Cont-SCID mice. Tumors formed from WT-DU145 cells but not from ICAM1-DU145 cells in Cont-SCID mice, while NKneut-SCID mice developed tumors comprising both cell types **(**Fig. 6 B**)**. Taken together, these results indicate that ICAM1 downregulation is an important factor underlying the high tumorigenic potential of PCa cells due to their ability to escape NK cell attack.

### ICAM1 expression is downregulated in malignant PCa patients

**Fig. 7:**
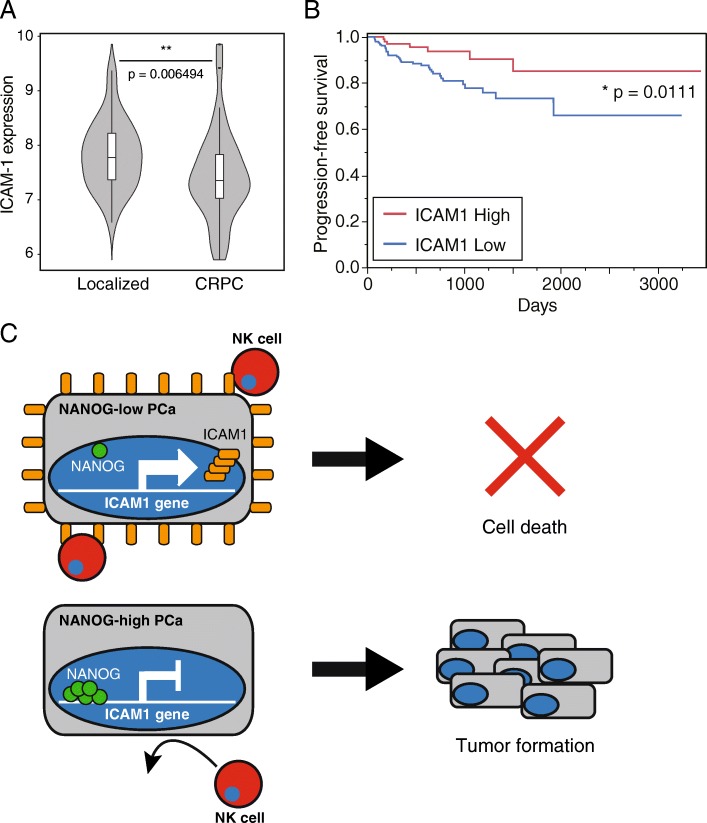
ICAM1 expression correlates with malignancy and recurrence in PCa patients (**a**) Correlation between ICAM1 expression levels and grades of PCa (localized and CRPC) malignancy in published microarray data. (**b**) Kaplan-Meier curves of the progression-free survival rates of TCGA PCa patients with high or low ICAM1 expression divided by the median. Log, log-rank test. (**c**) Model of escape system of PCa from NK cell attack. NANOG-low PCa cells are efficiently identified and destroyed by NK cells via ICAM1 expression. NANOG-high PCa cells downregulate ICAM1 expression and evade NK cell attak

Up to this point, our study indicated that PCa tumors in which ICAM1 expression was downregulated exhibited high malignancy in mouse model. In order to investigate whether same phenomenon is observed in PCa patients, the relationship between ICAM1 expression and patients with malignant PCa was analyzed. A NANOG-high/ICAM1-low-expressing population existed in a clinical sample of PCa tumors (Additional file 1: Figure S7), and the levels of ICAM1 expression between localized PCa and castration-resistant PCa (CRPC) were compared based on The Cancer Genome Atlas (TCGA) microarray data. ICAM1 was expressed at significantly lower levels in CRPC than in localized PCa **(**Fig. 7 A**)**. Moreover, we analyzed TCGA RNAseq data and assessed the recurrence rate of PCa tumors expressing high and low levels of ICAM1 after total prostatectomy. The PCa patients with low ICAM1 expression exhibited a significantly higher recurrence rate than PCa patients with high ICAM1 expression **(**Fig. 7 B**)**. These findings showed that the ICAM1 expression level is correlated with malignancy in PCa patients and that PCa patients expressing lower levels of ICAM1 show higher malignancy and higher recurrence rates than those expressing higher levels of ICAM1.

## Discussion

Cancer cells are attacked by mainly cytotoxic T lymphocytes (CTLs) and NK cells. CTLs take a long time to attack because some activation steps are required, such as phagocytosis of damaged cancer cells by antigen-presenting cells (APCs), migration of APCs from tumor tissue to lymph nodes, presentation of tumor-associated antigens (TAAs) or neoantigens to CTLs by APCs, activation of CTLs by some activation signals from APCs and migration of activated CTLs to tumor tissue [42]. Therefore, CTLs are unlikely to attack cancer cells during the early phase of tumorigenesis. On the other hand, NK cells are constantly trafficked throughout the entire body and are capable of immediately killing target cancer cells via adhesion. Therefore, because a few cancer cells are preferentially attacked by NK cells rather than CTLs in the absence of a tumor tissue-mediated immunosuppressive environment after tumorigenesis, these cancer cells should require their own system for escaping NK cells and allowing tumor tissue formation. In this study, PCa cells were shown to escape NK cell attack and exhibit tumorigenic potential via the NANOG-mediated downregulation of ICAM1 **(**Fig. 7 C**)**. Previous reports also showed NANOG-mediated ICAM1 expression regulation in hepatocellular carcinoma, however, ICAM1 was conversely upregulated by NANOG therein [43, 44]. Although it is unclear why such an opposite transcription regulation is modulated by NANOG between these cancers, the components of transcription factor complex might differ depending on the type of cancer. GN-DU145 cells effectively evaded NK cell attack via the downregulation of ICAM1 expression **(**Fig. 2 D**)**, and this escapability was ameliorated by forced ICAM1 expression (ICAM1/GN-DU145 cells) **(**Fig. 4 D**)**. ICAM1/GN-DU145 cells were able to form tumor tissue upon the neutralization of NK cells for 19 days after transplantation **(**Fig. 4 D**)**, and interestingly, ICAM1/GN-DU145-derived tumor growth was continuously promoted, like GN-DU145-derived tumor growth, after the discontinuation of NK cell neutralization. These findings suggest that cancer cells are not required to have individualized NK cell escape systems to promote tumor growth after the initiation of tumor tissue formation. Because established tumor tissue improves the immune suppressive environment therein, cancer cells could be protected from NK cell attack without requiring an escape system. On the other hand, residual cancer cells might escape not only NK cells but also CTLs during recurrence after the eradication of tumor tissue by cancer treatment because CTLs are already activated against tumor antigens. This study demonstrated that tumor tissue cells harvested from PCa patients exhibited high NANOG expression and low ICAM1 expression (Additional file 1: Figure S7), and PCa patients expressing low levels of ICAM1 had a higher risk of recurrence than those expressing high levels of ICAM1 after surgical cancer resection **(**Fig. 7 B**)**. ICAM1 was previously reported to contribute to the activation of not only NK cells but also CTLs [45, 46], and NANOG expression was shown to induce CTL resistance in cancer cells [47, 48]. Therefore, NANOG-mediated ICAM1 downregulation might protect PCa cells from attack by not only NK cells but also CTLs.

Several transcription factors contribute to the maintenance of CIC-like characteristics, which include high tumorigenic potential and some malignant phenotypes [30]. In particular, the upregulation of stem cell factors, e.g., NANOG, OCT4 and SOX2, are distinguishing characteristics of CIC-like cells, and these transcription factors are important for maintenance of the CIC-like phenotype [22]. NANOG is highly expressed in CIC-like PCa cells [31, 32], and our group has revealed that NANOG is important for the tumorigenesis of PCa [33]. Moreover, sphere-forming and highly malignant PCa cells, which are enriched CIC-like cells [16, 34], express NANOG at higher levels than adherent-cultured and low malignant PCa cells (Fig. 1 B, C **and** Additional file 1: Figure S2). Based on these results, we chose to focus on the role of NANOG in the resistance of PCa to NK cells, revealing that NANOG enhanced the tumorigenic potential of SCID mice by allowing escape from NK cells via repressing ICAM1 expression. It is possible that transcription factors other than NANOG, e.g., OCT4 and SOX2, may also be involved in ICAM1 downregulation in PCa. However, a previous report indicated that while NANOG bound to the ICAM1 promoter region in CIC-like hepatocellular carcinoma cells, OCT4 and SOX2 did not [44]. Therefore, ICAM1 suppression in CIC-like PCa cells is likely regulated by mainly NANOG, although SOX2 and OCT4 might control other immune escape mechanisms in CIC-like cells. Thus, PCa presumably acquires NK cell resistance and a CIC-like phenotype via several ICAM1 downregulation mechanisms, including NANOG.

Cancer cells utilize immune inhibitory molecules and actively suppress anticancer immunity. In particular, immune checkpoint stimulation by cancer cells has attracted attention in recent years. Various immune checkpoint molecules expressed in immune cells (e.g., PD-1, CTLA-4, TIGIT and TIM-3) inhibit activation and ultimately induce immune cell exhaustion by binding to respective ligands (e.g., PD-L1, CD80/CD86, PVR and Galectin-9) [49, 50]. Cancer cells express these immune checkpoint molecule ligands at high levels on their cell surface and induce T cell exhaustion for escape from CTL attack via the stimulation of immune checkpoints [51]. Moreover, cancer cells induce NK cell exhaustion by stimulating immune checkpoints (PD-1 and TIGIT) on NK cells [52–54]. Immune checkpoint stimulations are efficient mechanisms by which cancer cells escape anticancer immune responses, and highly tumorigenic cancer cells are thought to express these ligands at higher levels than other normal cancer cells to promote NK cell exhaustion. However, our results obtained herein indicated that GN-DU145 cells evade NK cell attack better than WT- and GFP-DU145 cells via the downregulation of ICAM1 but not via the upregulation of already-known inhibitory factors, including immune checkpoint ligands **(**Fig. 3 A **and** Additional file 1: Figure S5A**)**. These results suggest that although these NK cell inhibitory factors and immune checkpoint ligands might partially contribute to the ability of PCa cells to escape from NK cell attack, the CIC-like PCa-specific powerful NK cell resistance depends on downregulation of the NK cell activation factor. The expression of immune checkpoint molecules on NK cells is maintained at a low level in the inactivated state and increased after NK cell activation [55, 56]. When NK cells initially recognize newly born cancer cells, they likely still express immune checkpoint molecules at low levels due to inactivation. Therefore, CIC-like cells might effectively evade NK cell attack by downregulating NK cell activation factors rather than by upregulating immune checkpoint ligands.

Two main hypotheses, the clonal (or stochastic) model and the CIC (or hierarchical) model, have been proposed to explain the tumorigenesis mechanism [57, 58]. In the clonal model, almost all heterogeneous cancer cells are able to establish tumor tissue because most cancer cells have the ability to self-renew and differentiate into other types of cancer cells. In the CIC model, cancer cells are classified into two general groups: the CIC population and other differentiated cancer cells. CICs have the ability to self-renew and provide other differentiated cancer cells, while differentiated cancer cells are only capable of self-renewal. Therefore, only CICs and not the other differentiated cancer cells can form tumor tissue via the establishment of heterogeneous cancer cells. Previous reports have indicated that specific subpopulations of various cancer cells display highly tumorigenic potential in immunodeficient mice (SCID or NOD/SCID) [17–19, 59, 60]. Hence, these cancer cells have been identified as CICs, and the CIC hypothesis is thought to be a popular tumorigenic mechanism. CICs have specific characteristics, such as expressing various surface markers, intracellular proteins and transcription factors, e.g., CD133, CD44, ALDH1 and NANOG [17–19, 59, 60], and the most important CIC phenotype is their high tumorigenic potential. However, why and how CICs acquire high tumorigenic potential remain unknown. Here, we demonstrated that SP- and GN-DU145 cells, which exhibited high tumorigenic potential in SCID mice, were able to establish tumor tissue by escaping from NK cell attack due to suppression of ICAM1 expression **(**Fig. 1 A**)**. Moreover, (WT-) AD-DU145 cells, which had extremely low tumorigenic potential in normal SCID mice, were able to effectively establish tumor tissues in SCID mice due to the neutralization of NK cells **(**Fig. 1 A**)**. These results suggest that low-tumorigenic cancer cells maintain a sufficient ability to establish tumor tissue and that these cancer cells are killed by NK cells before tumor tissue formation because of an insufficient ability to escape. In other words, although most cancer cells are capable of forming tumor tissues, the presence or absence of tumorigenicity in cancer cells depends on whether the cells have the ability to escape from NK cell attack. Our findings serve as a basis for forming new tumorigenic mechanism hypotheses and suggest that NANOG-mediated ICAM1 downregulation is a key factor allowing the formation of tumor tissue via the evasion of NK cell attack.

## Conclusions

Herein, we showed that repression of ICAM1 is a critical mechanism by which cancer cells evade attack from NK cells during tumorigenesis. These results suggest a pivotal role of NANOG in establishing a gene expression profile for escaping the immune system.

## Supplementary information

**Additional file 1.** Supplementary methods and figures.

## Data Availability

The analyzed datasets and used materials in the current study are available from the corresponding author on reasonable request.

## Abbreviations

AD: Adherent-cultured
CICs: Cancer-initiating cells
Cont: Control
CRPC: Castration-resistant PCa
GFP: GFP-overexpressing
GN: GFP-NANOG-overexpressing
GN-P2A: GFP-P2A-NANOG
GS: Grison score
H3K27Ac: Histone H3 lysine 27 acetylation
ICAM1/GN: ICAM1-overexpressing GN-
ICAM1KO: ICAM1 knockout
NKneut: NK cell neutralized
PCa: Prostate cancer
SP: Sphere-forming
TAAs: Tumor-associated antigens
TCGA: The Cancer Genome Atlas
WT: Wild type
