# Supplementary material for: NANOG helps cancer cells escape NK cell attack by downregulating ICAM1 during tumorigenesis
Source: J Exp Clin Cancer Res. 2019 Oct 16;38:416. doi: 10.1186/s13046-019-1429-z (PMC6796413; doi:10.1186/s13046-019-1429-z)

Supplementary methods

Fluorescence immunostaining

Human primary prostate cancer (hPCa) tissues obtained from Osaka University Hospital were fixed with formaldehyde, embedded in paraffin and sectioned into 5-μm-thick slices. Tissue sections were deparaffinized with 100% xylene, rehydrated with a gradient series of EtOH (100%, 90%, 80% and 70%), permeabilized with 0.1% Triton X-100 in PBS and blocked with 10% normal goat serum (NGS) (Sigma, G9023). NANOG and ICAM1 were respectively stained with an anti-NANOG Ab (Alexa Fluor 647; BD Pharmingen, CA, USA; 561300) and an anti-ICAM1 Ab (FITC; Sino Biological, Beijing, China; 10346-MM01-F). Isotypes (BD Pharmingen; 557714, Biolegend, CA, USA; 400108) were used as controls. After staining, the sections were mounted with ProLong Gold antifade reagent, which included DAPI (Invitrogen). The stained sections were observed on a confocal microscope (Nikon instech Co., Kanagawa, Japan), and the images were analyzed using NIS-Elements AR3.2 software (Nikon instech).

NK cell-like lysis activity assay of MTA cells

K562 cells ($5x10^5$ cells) were stained with PKH67 and then cocultured with MTA or M1 cells (macrophage cell line) ($5x10^5$ cells) for 48 hours. Cocultured cells were stained with TO-PRO-3 iodide (642/661) (Invitrogen) and fixed with 1% paraformaldehyde. Attacked K562 cells (PKH67+/TO-PRO-3+ cells) were analyzed on a FACSCant II instrument.

FACS analysis of NK cell resistance

DU145 ($1x10^5$ cells) and MTA ($5 x10^5$ cells) were co-cultured in 96-well U bottom plates for 6 hours. Human NK cell was isolated by Ficoll-Paque Premium (GE Healthcare) and human NK cell isolation kit (Biolegend). DU145 ($1x10^5$ cells) and NK cell ($9 x10^5$ cells) were co-cultured in 96-well U bottom plates for 24 hours. Co-cultured cell mixture was stained by Brilliant Violet 421 anti-human CD56 antibody (Biolegend; #318328), and then, by APC-AnnexinV (Biolegend; #640941). Attacked DU145 cells (CD56- /AnnexinV+ cells) were analyzed on a FACSCant II instrument.

Quantification of miRNA expression

miR-296-3p expression level was quantified by TaqMan microRNA Assays (ThermoFisher Scientific) for miR-296-3p (assay ID: 478790_mir).

Plasmid construction

The P2A sequence was inserted between the EGFP sequence and the NANOG sequence of GFP-NANOG by PCR, and GFP-P2A-NANOG cDNA was introduced into the CAGIpuro vector.

Supplementary figure legends

Supplementary figure S1

(**A**) Photos of AD-DU145 and SP-DU145 cells. (**B**) Experimental model of AD- and SP-DU145 cell intradermal transplantation into SCID mice. (**C**) AD- and SP-DU145 cells were transplanted onto SCID mice (left flank: AD, right flank: SP). The respective tumor volumes were measured for up to 60 days after the transplantation (n=4, *P < 0.05, **P < 0.01, ***P < 0.005, Student's t test). (**D**) The strategy for assaying tumor formation in SCID mice with or without NK cell neutralization. **(1),** An anti-asialo GM1 antibody or control IgG was intraperitoneally administered to SCID mice, and **(2),** DU145 cells and their derivatives were intradermally transplanted onto the backs of these mice the following day. **(3),** The anti-asialo GM1 antibody or control IgG was intraperitoneally administered nine times on days 0, 1, 2, 4, 6, 9, 12, 15 and 19 after cancer cell transplantation. Tumor volume measurements began 30 days after the transplantation.

Supplementary figure S2

Immunofluorescence staining of NANOG in PCa patient-derived tumor sections (red: NANOG, blue: DAPI). Arrowheads indicate NANOG signals.

Supplementary figure S3

(**A**) Western blot analyses of WT-, GFP- and GN-PC3 cells. (**B**) The proliferation rates of PC3 cells (WT, GFP, and GN) were compared (n=3, Tukey-Kramer test). (**C**) NK cell-like lysis activity assay of MTA cells. K562 cells were cocultured with

MTA or M1 cells, and the attacked K562 cells were stained with TO-PRO-3 iodide. The red line shows K562 cells that were not cocultured, and the blue line shows K562 cells that were cocultured with MTA or M1 cells. (**D**) The cell viabilities of PC3 cells (WT, GFP, and GN) were measured after performing the NK cell resistance assay in vitro (n=3, *P < 0.005, **P < 0.001, Tukey-Kramer test). (**E**) MTA-treated AnnexinV+ DU145 cells were measured by FACS analysis. (n=3, **P < 0.001, Studend t test). (**F**) NK cell-treated AnnexinV+ DU145 cells were measured by FACS analysis. (n=3, *P < 0.05, Studend t test).

Supplementary figure S4

(**A** and **B**) Validation of RNAseq and ChIPseq data. Correlation of RNAseq data (**A**) and ChIPseq data (**B**) between replicates.

Supplementary figure S5

(**A**) RNAseq analyses of WT-, GFP-, and GN-DU145 cells. The expression levels of immune checkpoint ligands were compared among these cells. (**B**) Western blot analyses of WT-, GFP- and GN-22Rv1 cells. (**C**) Quantification of microRNA (miR-296-3p) expression in WT-, GFP-, and GN-DU145 cells (n=3, **P < 0.01, Tukey-Kramer test). (**D**) ICAM1 expression was detected in GFP-, GN- and GN-P2A-DU145 cells by western blot.

Supplementary figure S6

(**A**) ICAM1 and NANOG expression in WT- and ICAM1KO-DU145 cells was compared. (**B**) The proliferation rates of WT- and ICAM1KO-DU145 cells were

compared (n=3, Student's t test). (**C**) ICAM1 expression in WT- and ICAM1-DU145 cells was compared. (**D**) The proliferation rates of WT- and ICAM1-DU145 cells were compared (n=3, Student's t test).

Supplementary figure S7

Immunofluorescence staining of ICAM1 and NANOG in PCa patient-derived tumor sections (green: ICAM1, red: NANOG, blue: DAPI). The fluorescence intensities of ICAM1 and NANOG signals were measured along a line from (i) to (ii).

Supplementary Figure S1

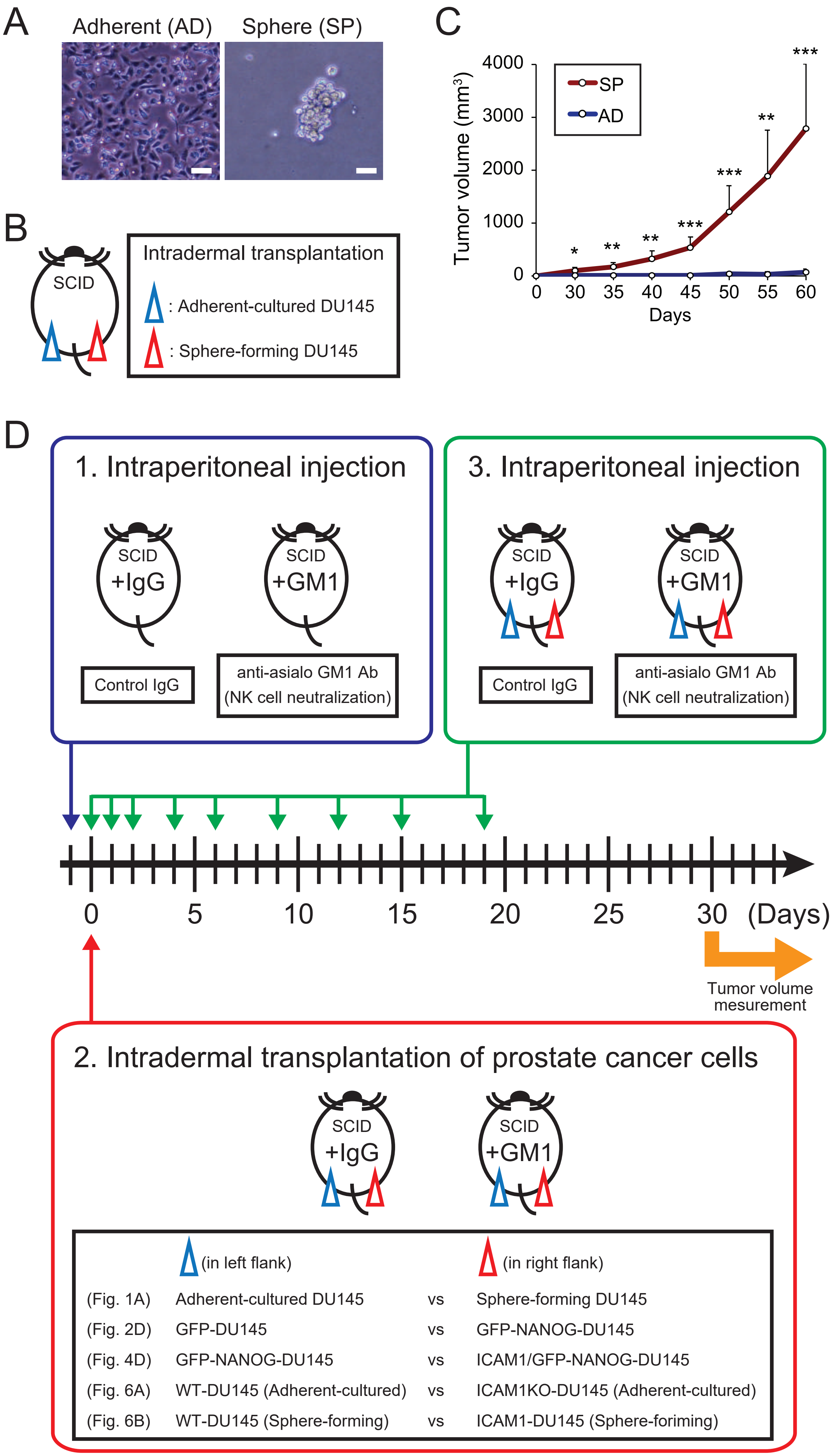

Supplementary Figure S2

Grison score 6 (3, 3) | Grison score 9 (4, 5)

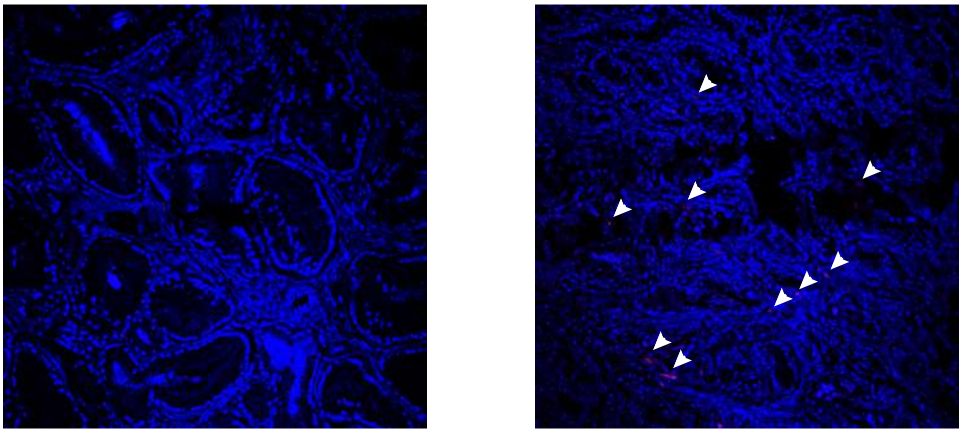

Supplementary Figure S3

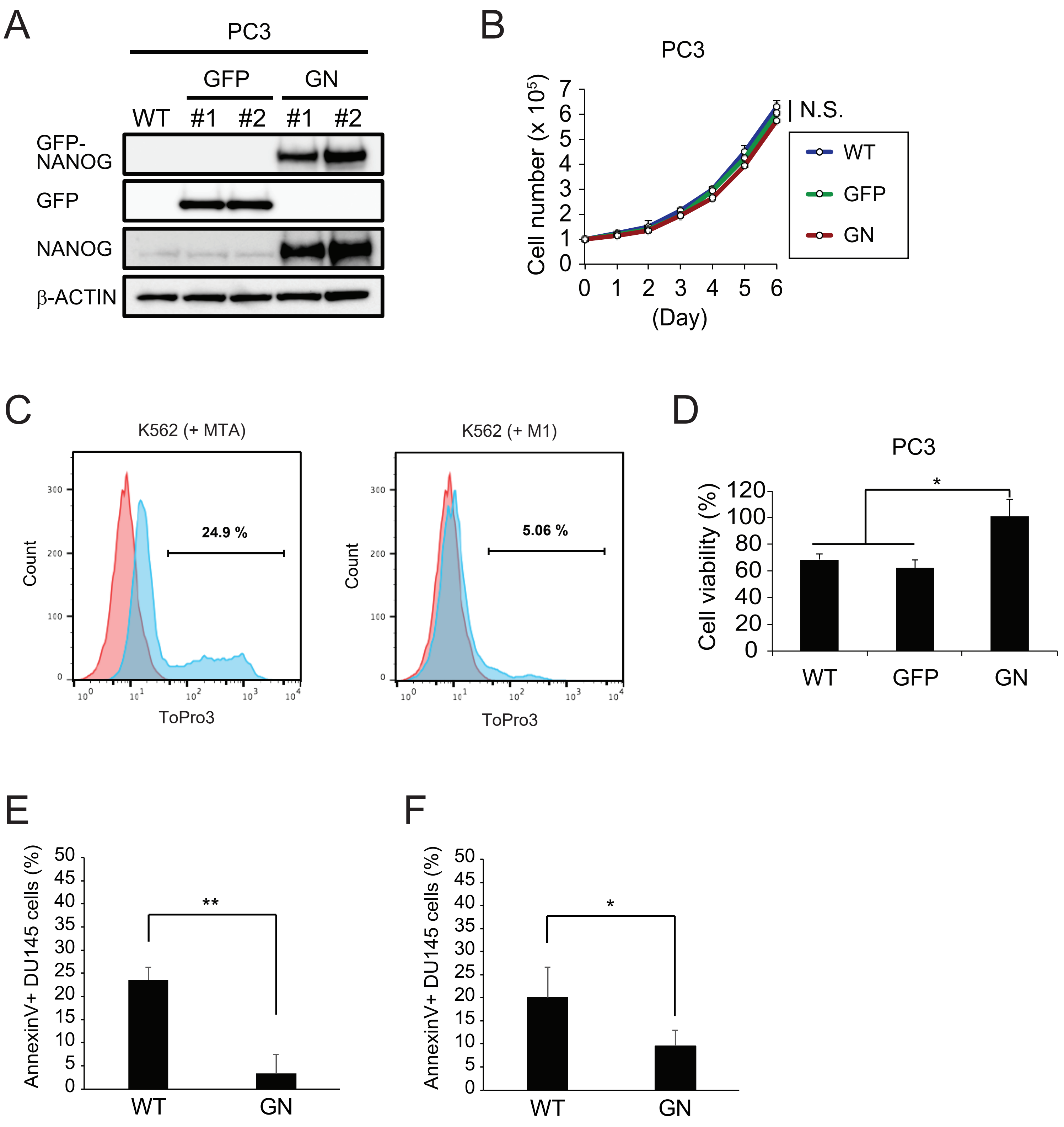

Supplementary Figure S4

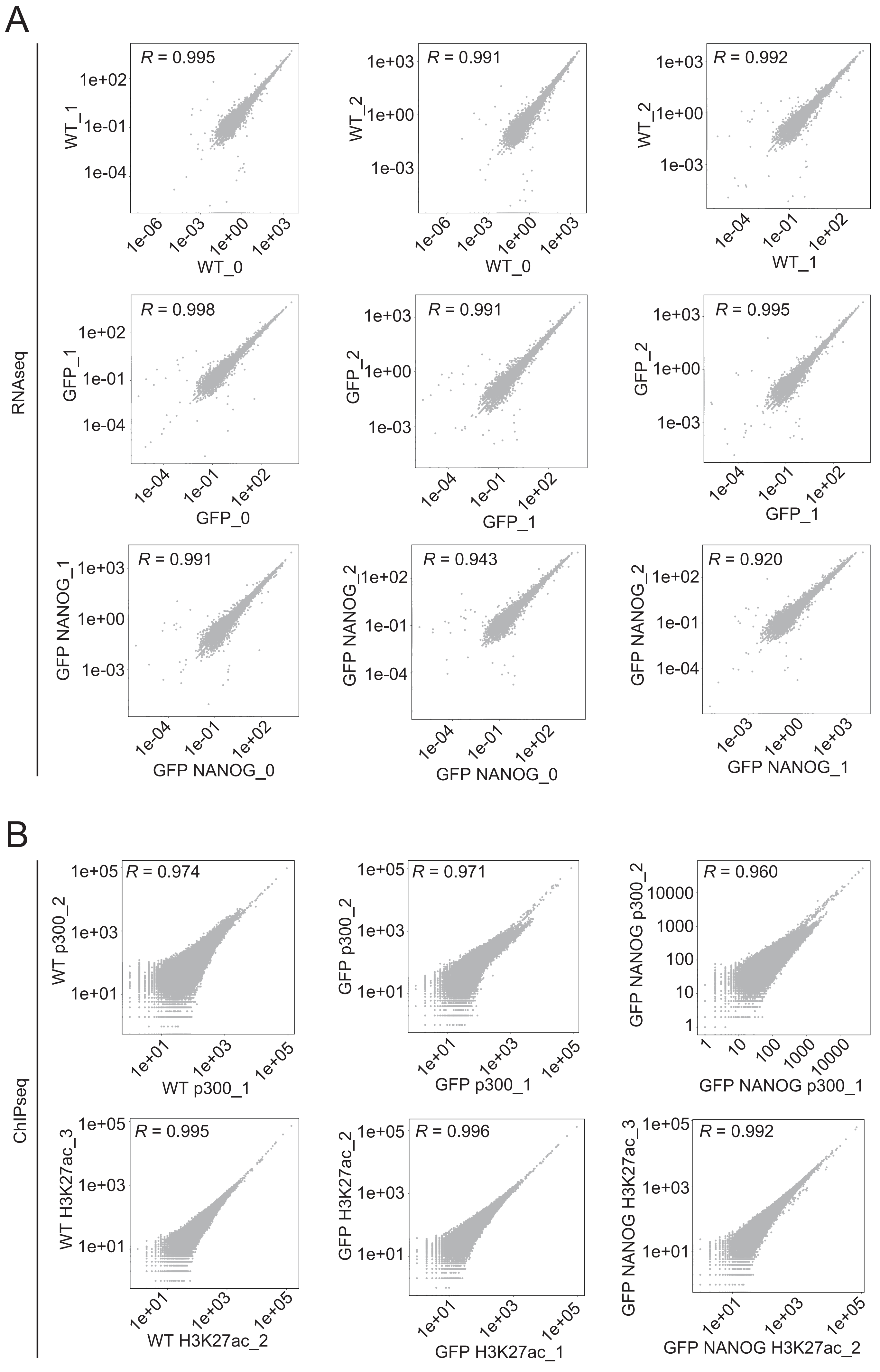

Supplementary Figure S5

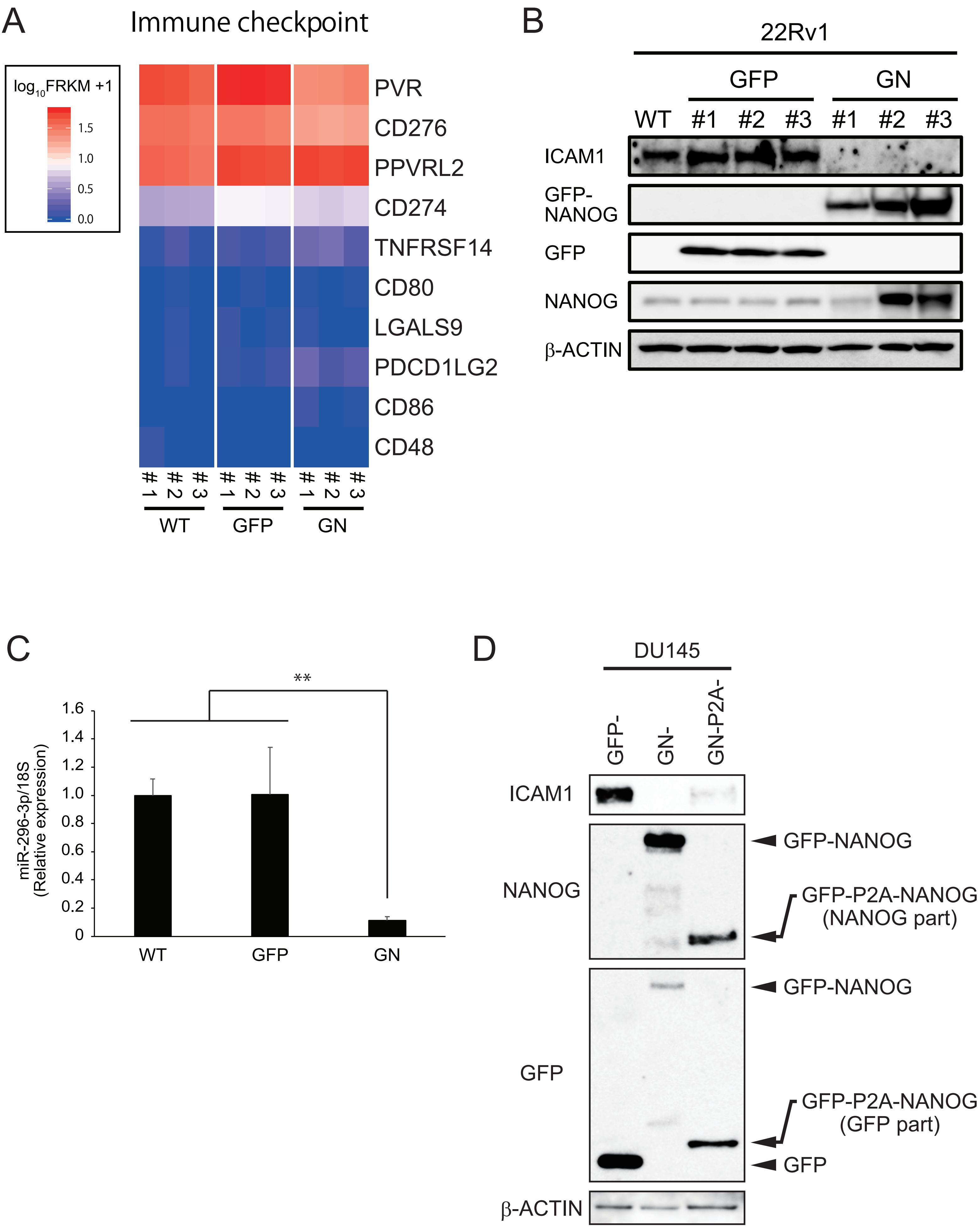

Supplementary Figure S6

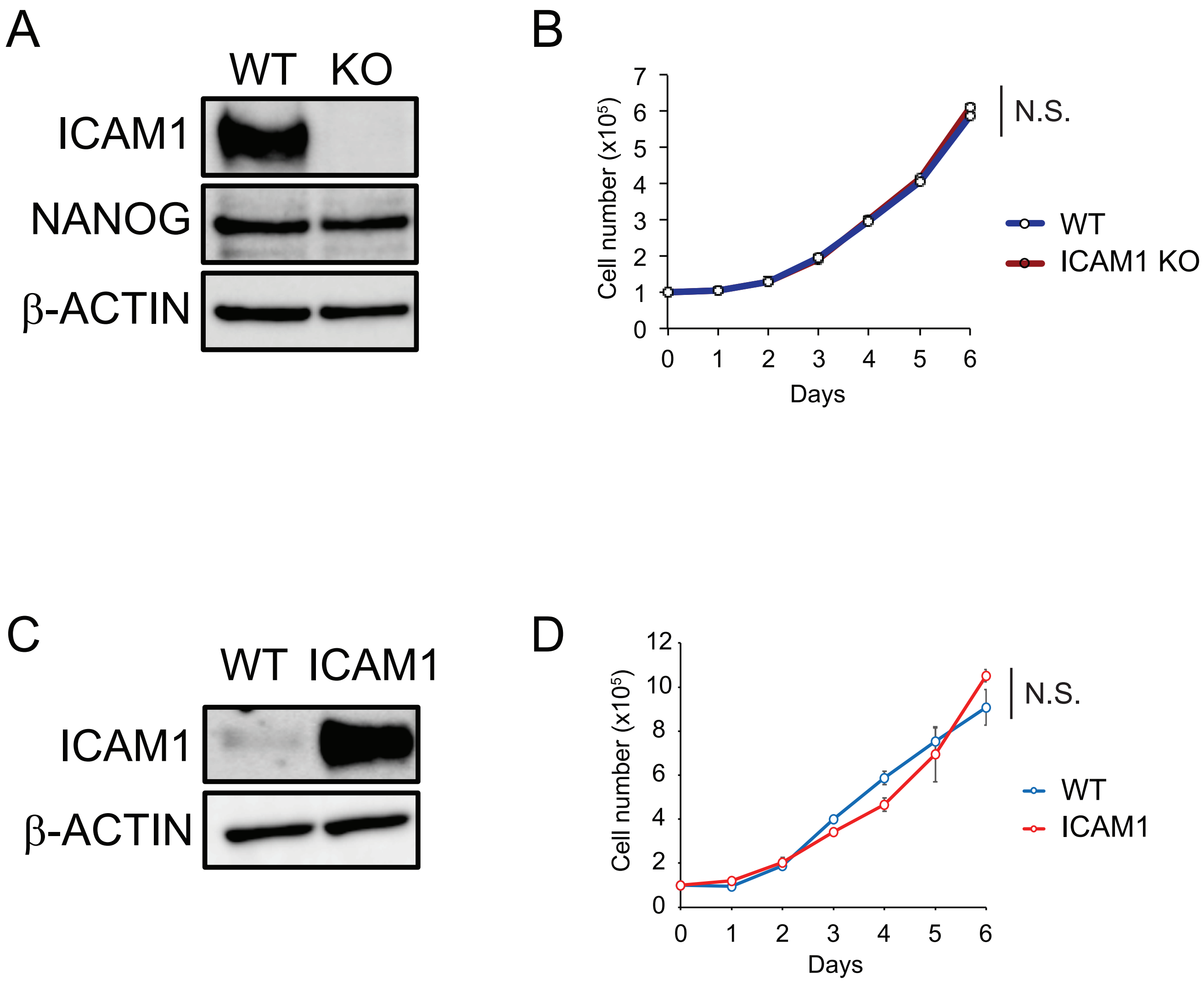

Supplementary Figure S7

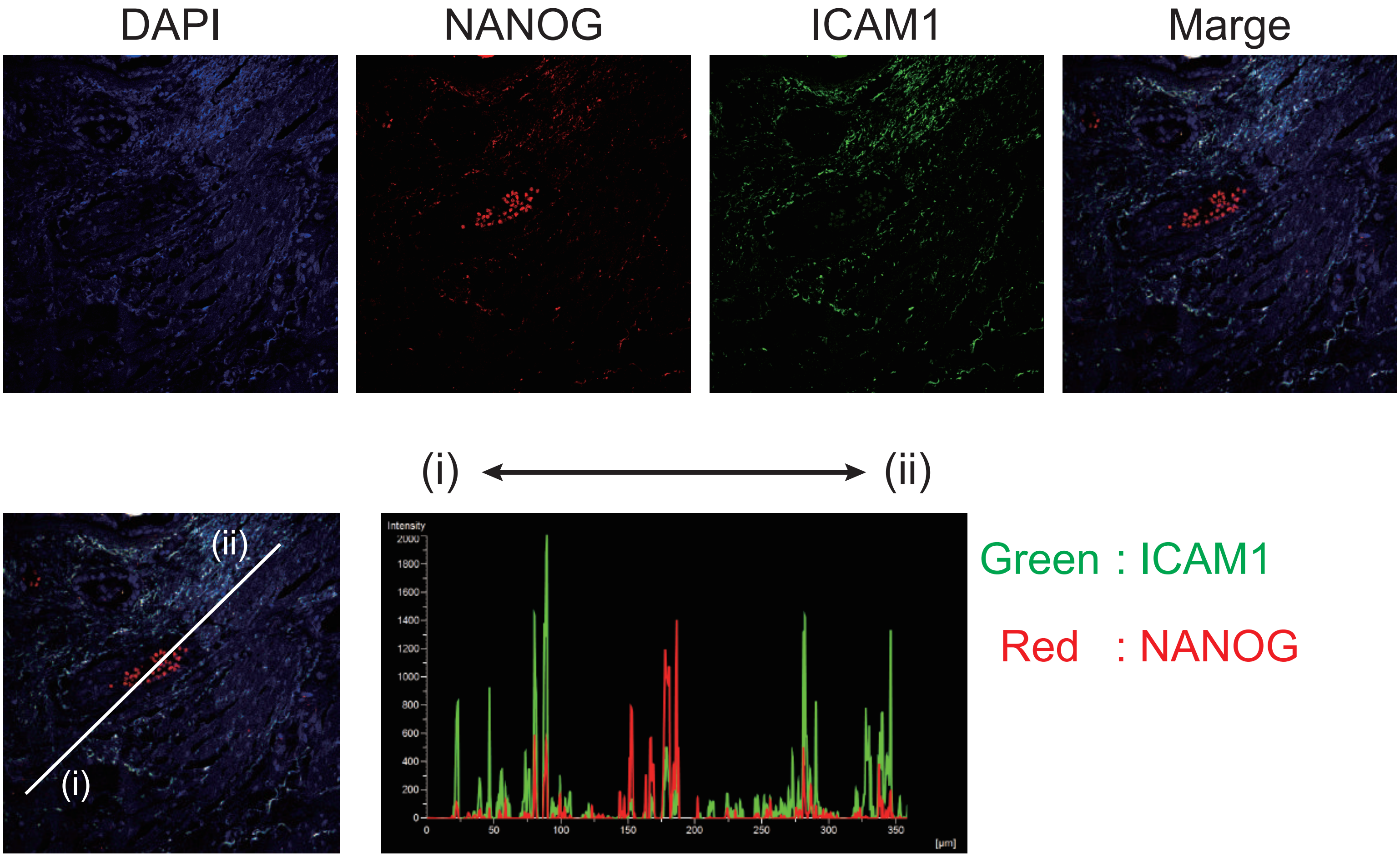

Supplement: Supplementary file 1 — Additional file 1. Supplementary methods and figures. [file 13046_2019_1429_MOESM1_ESM.pdf]
